# Incidence and predictors of *Escherichia coli* producing extended-spectrum beta-lactamase (ESBL-Ec) in Queensland, Australia from 2010 to 2019: a population-based spatial analysis

**DOI:** 10.1017/S0950268822001637

**Published:** 2022-10-26

**Authors:** Weiping Ling, Angela Cadavid-Restrepo, Luis Furuya-Kanamori, Patrick N. A. Harris, David L. Paterson

**Affiliations:** 1 Faculty of Medicine, University of Queensland, UQ Centre for Clinical Research, Herston, Brisbane, Australia; 2 Faculty of Medicine, University of Queensland, School of Public Health, Herston, Brisbane, Australia; 3 Central Microbiology, Pathology Queensland, Royal Brisbane & Women's Hospital, Herston, Brisbane, Australia; 4 ADVANCE-ID, Saw Swee Hock School of Public Health, National University of Singapore, Singapore

**Keywords:** Antibiotic resistance, community, *E. coli*, epidemiology, ESBL

## Abstract

The dissemination of *Escherichia coli* producing extended-spectrum beta-lactamase (ESBL-Ec) is evident in the community. A population-based spatial analysis is necessary to investigate community risk factors for ESBL-Ec occurrence. The study population was defined as individuals with ESBL-Ec isolated in Queensland, Australia, from 2010 to 2019. Choropleth maps, global Moran's index and Getis-Ord Gi* were used to describe ESBL-Ec distribution and identify hot spots. Multivariable Poisson regression models with or without spatially structured random effects were performed. A total of 12 786 individuals with ESBL-Ec isolate were identified. The crude incidence rate increased annually from 9.1 per 100 000 residents in 2010 to 49.8 per 100 000 residents in 2019. The geographical distribution of ESBL-Ec changed from random to clustered after 2014, suggesting presence of community-specific factors that can enhance occurrence. Hot spots were more frequently identified in Outback and Far North Queensland, future public health measures to reduce transmission should prioritise these communities. Communities with higher socioeconomic status (RR = 0.66, 95% CI 0.55–0.79, per 100 units increase) and higher proportion of residents employed in the agricultural industry (RR = 0.79, 95% CI 0.67–0.95, per 10% increase) had lower ESBL-Ec incidence. Risk factors for occurrence appear differential between remote and city settings and this should be further investigated.

## Introduction

The community spread of *Escherichia coli* producing extended-spectrum beta-lactamase (ESBL-Ec) is notable in the last decade. ESBL-Ec are extra-intestinal pathogenic bacteria that predominantly cause urinary tract infections while development of either primary or secondary bloodstream infections are also common. Although carriage may not present with clinical implications, several high-risk groups, such as older people and immunocompromised patients, may be at elevated risk of life-threatening infections. It was observed that the odds of mortality in patients with ESBL-Ec infection were 70% higher than patients with non-ESBL-Ec infection [1]. In Australia, of all mortality cases from *E. coli* bloodstream infections in 2019, ESBL-Ec were detected in 18.3% of the cases, of which more than half were of community-onset [2].

Although previously treated as a healthcare-associated pathogen, an eight-fold increase in global ESBL-Ec carriage rate in healthy individuals was reported between 2003 and 2018 [3].

The widespread emergence of ESBL-Ec outside of hospital settings can be attributed to several reasons, including the establishment of a successful clonal group, namely *E. coli* ST131 that is highly associated with ESBL carriage [4]. The presence of long-term ESBL-Ec carriers in the community and importation of multidrug-resistant pathogens from travellers also increase the risk of ESBL-Ec exposure for general community dwellers [5, 6]. A modelling study from the Netherlands had documented that 67% of ESBL-Ec transmission in the community can be attributed to human-to-human spread [7]. Individual risk factors for ESBL-Ec infection such as old age, underlying disease and recent history of antibiotics use and hospitalisation are well-documented [8]. The risk factors in the community, that can promote inter-human spread, are still not extensively studied. Nonetheless, several observational studies have postulated that contact with farm animals [7, 9, 10], retail meat for consumption [7, 11], and household composition and density are predictors of ESBL-Ec acquisition in the community setting [12–14]. A high prevalence of migrants from ESBL-Ec endemic countries, such as Southeast Asia [3, 8, 15], may also introduce ESBL-Ec into the host country [16], and thereby increase the risk of human transmission.

The availability of population-based studies investigating community risk factors of ESBL-Ec spread is still scarce. Moreover, current population-based studies do not account for the spatial relationship between neighbouring communities and as such, do not consider influence from neighbouring features. With ESBL-Ec increasingly detected in the community, the need for spatial population-based epidemiological studies to understand its distribution pattern and risk factors is warranted. The higher risk of hospitalisation and mortality associated with ESBL-Ec infection warrants these public health investigations to allow for mitigation of ESBL-Ec spread. With significant increases in the occurrence of *E. coli* isolates resistant to ceftriaxone and ceftazidime detected in Australian communities [2], this population-based study aimed to describe the geographical patterns of ESBL-Ec in Queensland over a ten-year period, and to identify the predictors of ESBL-Ec transmission in the community.

## Methods

This manuscript followed the Strengthening the Reporting of Observational Studies in Epidemiology (STROBE) Statement for recommended reporting guidelines [17]. The study protocol was reviewed and approved by the University of Queensland Human Research Ethics Committee (2020001388). As this is a population-based ten-year study using de-identified data, a waiver of consent was obtained for its low and negligible risk nature.

### Study design, setting and participants

This study was based on an ecological design. The study population was defined as all Queensland, Australia, residents with ESBL-Ec isolates from 2010 to 2019. This included both asymptomatic and sick residents with detected ESBL-Ec isolate. Queensland is in North-Eastern Australia, spans approximately 1.7 million km^2^ of land area and includes costal, mountainous as well as arid regions. It is the second largest state in Australia with 5.2 million residents from 442 postal areas in 2021. Most of Queensland residents reside in the state capital, Brisbane, as well as in other coastal cities including Gold Coast, Sunshine Coast, Cairns and more.

### Outcome data

The study data were extracted from AUSLAB (PJA Solutions Pty Ltd) database, Pathology Queensland, which is the pathology laboratory that records and processes all microbiological samples collected from public healthcare facilities in Queensland. The public healthcare services are distributed and managed across 15 different regional and metropolitan Hospital and Health Services in Queensland (Supplementary Fig. S1). There are 36 laboratories from Pathology Queensland to service these public healthcare facilities. Pathology Queensland determines ESBL production using EUCAST Breakpoint Tables (currently version 11). The combination disc test is used to confirm ESBL-Ec isolates that had initial susceptibility testing performed on Vitek2 (BioMerieux), demonstrating resistance to ceftriaxone or ceftazidime. Data were collected from 2010 to 2019. Only unique individuals were included in the study sample and the year of ESBL-Ec acquisition was documented as their first isolate detected during the study period. Individuals with recorded postal code outside of Queensland were excluded. For each identified isolate, residential postal area, date of specimen collection, gender, age and specimen type were also recorded. The acquisition nature (community- or healthcare-acquired) could not be determined with our dataset.

### Explanatory variables

The population and demographic profile per postal area were collected from Australian Bureau of Statistics (ABS) [18], using census data from 2011 and 2016. Because population counts were not available for the other years, estimates were obtained by aggregating population counts from smaller administrative area (Statistical Area 1) that were publicly available from Queensland Government [19]. Community demographic profile for each Queensland postal area pertaining to population density, median age, proportion of males, indigenous people, migrants, rental properties, residents employed in health care services and residents employed in agricultural industry, housing composition and density (average number of children per family, average residents per house and average residents per bedroom) and index of relative socioeconomic disadvantage (IRSD), education and income were collected and described in Supplementary Table S1. Queensland postal area-level shapefiles (boundary map) were downloaded from ABS [20].

### Statistical analysis

#### Distribution of ESBL-Ec and hot spot analysis

The annual ESBL-Ec incidence rate per 100 000 residents was calculated using number of cases identified in each year as numerator and total number of Queensland residents in each year as denominator. The annual standardised morbidity ratio (SMR) of each postal area was calculated using observed number of ESBL-Ec cases divided by expected number of ESBL-Ec cases, where expected frequency was derived from annual Queensland incidence rate multiplied by population of each postal area. The SMR values were used to create annual choropleth maps of the spatial distribution of ESBL-Ec cases across Queensland. Next, hot spot analysis was performed by calculating global Moran's index to determine overall spatial autocorrelation. The assumption of inverse distance spatial relationship among postal areas, where closer neighbours were assigned higher weightage as compared to farther neighbours, was applied. The ESBL-Ec spatial pattern was determined as clustered if z-score fell above 1.96, as dispersed if z-score fell below −1.96 and as random if otherwise. Next, local Getis-Ord Gi* was performed to visually identify hot or cold spots of statistical significance. The assumption of inverse distance spatial relationship among postal areas was similarly applied here. There was no threshold distance applied to assume relationship among all postal areas. Distance method was set as Euclidean. To assess differences in results based on spatial relationships of features, a repeated Getis-Ord Gi* was performed using the default threshold distance (derived as 218 kilometers), where every feature had at least one other neighbour. All spatial mapping and hotspot analysis were performed on ArcGIS Pro.

#### Regression modelling with and without spatial parameter

Three postal areas with no residing population were excluded from the spatial analysis. The outcome was defined as all ESBL-Ec cases identified in 2016 per postal area and the predictors were demographic variables collected from 2016 census. All pre-determined variables were checked for correlation (*r* > 0.7 or *r* ≤ 0.7) and collinearity (variance inflation factor >5). In the event where high correlation or collinearity was detected between two variables, the variable that returned a lower Akaike information criterion score in a univariate Poisson regression model was selected. Following these model diagnostic tests, the variables tertiary education and median personal income were dropped from the model. Selection of demographic variables was performed on STATA/SE 16.1 and the univariate Poisson regression analysis of selected variables is documented in Supplementary Table S2.

Three Poisson regression models were developed in WinBUGS version 1.4.3 (MRC Biostatistics Unit 2008) using a Bayesian framework. The first model included only the unstructured random effects (*μ*) at postal area level, the second model included the spatially structured random effects (*s*) at postal area level and the third convoluted model (*μ* + *s*) included both unstructured and spatially structured random effects at postal area level. The convoluted Poisson model followed the following equation –
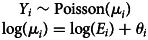


*θ_i_* = *α* + *β*_1_[population density]*_i_* + *β*_2_[median age]*_i_* + *β*_3_[proportion of males]*_i_* + *β*_4_[proportion of indigenous residents]*_i_* + *β*_5_[proportion of migrants]*_i_* + *β*_6_[average children per family]*_i_* + *β*_7_[average residents per house]*_i_* + *β*_8_[proportion of rented households]*_i_* + *β*_9_[proportion of residents employed in health care services]*_i_* + *β*_10_[proportion of residents employed in agricultural industry]*_i_* + *β*_11_[average residents per bedroom]*_i_* + *β*_12_[IRSD]*_i_* + *μ_i_* + *s_i_*,

where *Y*_i_ refers to the observed cases at the *i* postal area, *E_i_* refers to the expected cases at the *i* postal area and *θ* is the mean log relative risk. The random effects (*μ*, *s*) assumed normal prior distributions. The precision of random effects (*μ*, *s*) assumed gamma prior distribution of 0.5 and 0.0005. The intercept (*α*) assumed continuous uniform prior distribution with infinity bounds. The coefficients of covariates (*β*) assumed normal prior distributions with mean = 0 and precision = 0.001. Each covariate (*x*) was standardised using *z* = (*χ*  − population mean/population standard deviation). To model the spatial autocorrelation effects on WinBUGS, a conditional autoregressive (CAR) prior structure was used. Spatial relationships between postal code areas were determined using an adjacency weights matrix, which assigns weight = 1 to postal areas with shared boundary and weight = 0 if otherwise. The initial values were specified as 0 for the intercept and covariates, and as 0.5 for the precision of the random effects.

The zero-inflated Poisson regression models were also assessed in WinBUGS to determine the best model fit for this dataset. Models returning lower deviance information criterion (DIC) values were considered better. The Poisson models consistently returned lower DIC than the zero-inflated Poisson models and as such, Poisson regression was selected for this analysis.

For each model tested, a random seed of 5000 was set and an initial burn-in of 1000 iterations was made and discarded. Convergence of parameters was monitored with every subsequent 10 000 iterations, based on visual inspection of autocorrelation and history plots. Each of the Poisson regression model converged after 20 000 iterations. After convergence was met, another 20 000 iterations were run for the posterior distribution. The DIC value, posterior mean, standard deviation and 95% credible interval (95% CI) of each parameter, were stored and used for analysis. Statistical significance was set as 0.05.

Sensitivity analyses were conducted according to these model specifications and assumptions, using demographic data collected from the 2011 census and outcome defined as all ESBL-Ec cases identified in 2011.

## Results

A total of 12 786 unique patients with ESBL-Ec from 369 postal areas were recorded over the ten-year period. This included 7884 (61.7%) patients with ESBL-Ec isolates detected from urinary specimens only, 380 (3.0%) patients with ESBL-Ec isolates detected from blood specimens only and 733 (5.7%) patients with ESBL-Ec isolates from both urine and blood specimens. There were 2867 (22.4%) patients with no positive clinical culture but ESBL-Ec was isolated from their faecal specimens or rectal/anal swabs. The patients were predominantly female (63.1%). The mean age was 58.5 years (s.d. = 25.0), with 995 patients (7.8%) aged less than 18 years, 5483 (42.9%) aged between 18 and 65 years, and 6308 (49.3%) aged above 65 years.

### Distribution of ESBL-Ec and hot spot analysis

**Fig. 1.**
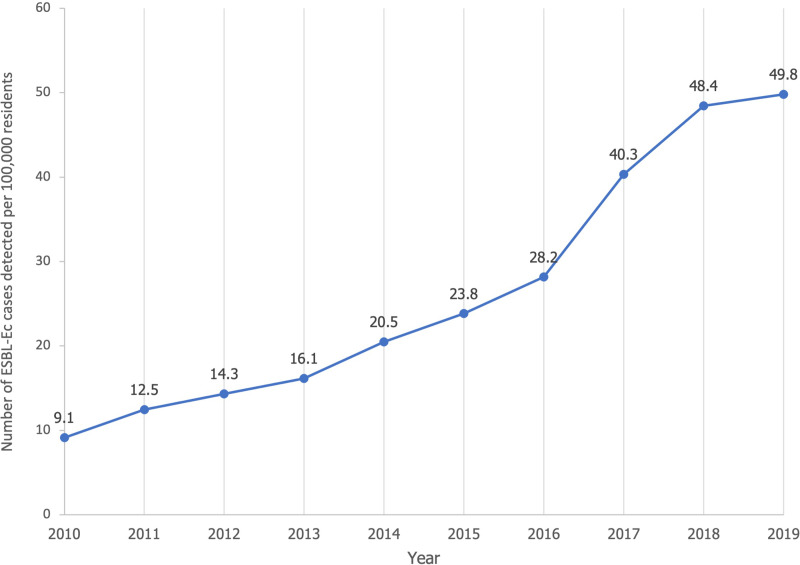
Annual incidence rate of ESBL-Ec per 100 000 residents in Queensland from 2010 to 2019.

**Fig. 2.**
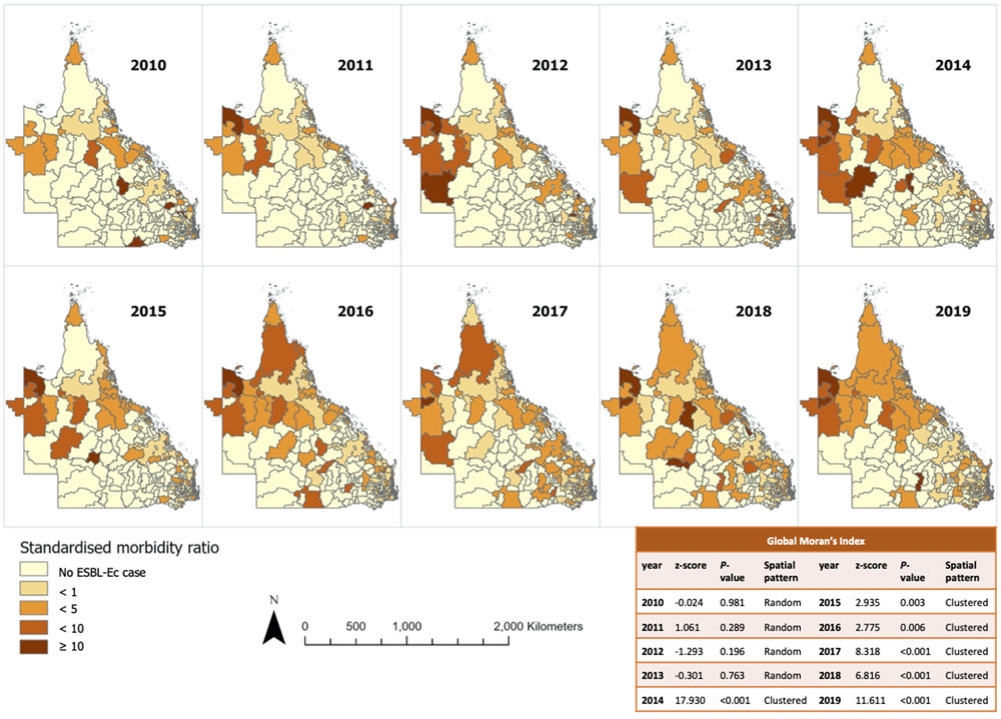
Annual SMR of ESBL-Ec incidence per postal area and overall geographical distribution across Queensland.

The incidence rate increased annually from 9.1 per 100 000 residents in 2010 to 49.8 per 100 000 residents in 2019 (Fig. 1), with the sharpest rise detected between 2016 and 2018. Annual choropleth maps of ESBL-Ec SMR are presented in Figure 2. Areas with higher-than-expected number of cases (SMR>1) were more prevalent from 2014 onwards. Using global Moran's index, the distribution of ESBL-Ec cases across Queensland was random between 2010 and 2013 (*P* > 0.05) but became significantly clustered between 2014 and 2019 (*P* < 0.05) (Fig. 2).

**Fig. 3.**
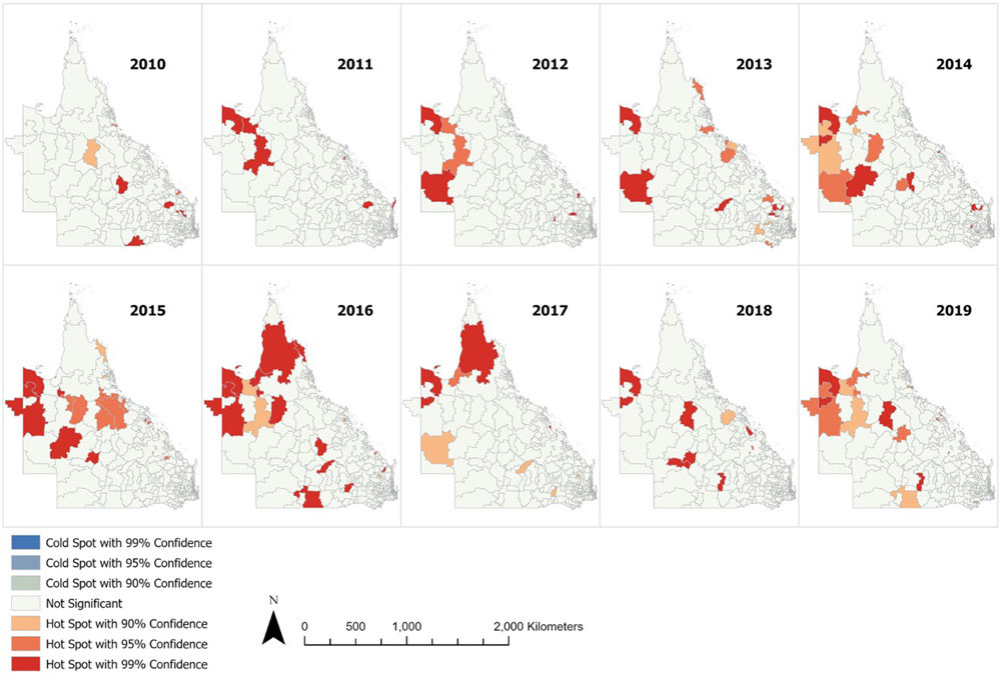
Annual ESBL-Ec hot spots identified in Queensland.

The ESBL-Ec hot spots of at least 90% confidence are detailed in Figure 3 (with focused map of state capital Brisbane in Supplementary Fig. S2). The hot spots identified using either default (218 kilometres) or no threshold distance were identical. Hot spots were more frequently detected from 2014 to 2016 inclusive, with the highest number of hot spots observed in 2016. It appeared that hot spots were more prevalent in remote Outback and Far North Queensland. Sporadic hot spots were also detected in the outer regional areas throughout the study period. A few coastal and inner regional hot spots that were detected in 2013 disappeared in the following years. The hot spots identified within Brisbane city were scarce and sporadic as compared to regional and remote areas. There was no cold spot of statistical significance identified.

### Predictors of ESBL-Ec incidence

The demographic characteristics of the remaining 439 postal areas in 2016 is summarised in Table 1. A total of 1364 patients with ESBL-Ec was detected in 2016. In the multivariable analysis, the Poisson model with spatially structured random effects showed the best performance (DIC = 1271.20). Using posterior means from this model, communities with higher IRSD score (RR = 0.66, 95% CI 0.55–0.79, per 100 units increase), which translates to better socioeconomic status, had significantly lower ESBL-Ec incidence (Table 2). Communities with higher proportion of residents being employed in the agricultural industry (RR = 0.79, 95% CI 0.67–0.95, per 10% increase) was also a significant predictor of lower ESBL-Ec incidence. A higher proportion of Aboriginal and Torres Strait Islander residents in the community was the only predictor for higher ESBL-Ec incidence (RR = 1.13, 95% CI 0.96–1.33, per 10% increase), although this observation was not statistically significant.

**Table 1.** Summary of community demographic factors in 439 Queensland postal areas with residents in 2016

Demographic variable	Mean (s.d.)
Number of people per km squared	490.0 (901.4)
Median age (year)	40.6 (7.0)
Proportion of male (%)	50.8 (3.1)
Proportion of Aboriginal and Torres Strait Islander (%)	5.6 (10.4)
Proportion of residents born in Australia (%)	75.3 (10.7)
Proportion of unemployed residents (%)	7.0 (4.0)
Average number of children per family	0.7 (0.2)
Average number of residents per house	2.5 (0.3)
Proportion of rented households (%)	32.1 (14.7)
Proportion of residents employed in health care services (%)	10.5 (4.2)
Proportion of residents employed in agricultural industry (%)	14.1 (18.0)
Average number of people per bedroom	0.8 (0.1)
IRSD	978.6 (74.3)

**Table 2.** Multivariable spatial analysis of demographic predictors and ESBL-Ec incidence in 2016, with comparison of results across 3 different models

Year 2016	Spatially unstructured random effects model (*s* + *μ*)	Spatially structured model (*s*)	Unstructured random effects model (*μ*)
**Demographic variable**	**Relative risk (95% CI)**		
Number of people per km squared (per 1000 people increase)	0.93 (0.80–1.08)	0.94 (0.81–1.09)	0.96 (0.84–1.09)
Median age (per 1 year increase)	0.98 (0.95–1.00)	0.98 (0.95–1.00)	**0.97** (**0.94–0.99)**
Proportion of male (per 10% increase)	0.95 (0.56–1.60)	0.95 (0.56–1.59)	1.19 (0.70–1.97)
Proportion of Aboriginal and Torres Strait Islander (per 10% increase)	1.12 (0.95–1.33)	1.13 (0.96–1.33)	1.20 (1.01–1.43)
Proportion of residents born in Australia (per 10% increase)	1.00 (0.86–1.17)	1.00 (0.86–1.16)	0.96 (0.84–1.10)
Average number of children per family (per 0.1 unit increase)	0.95 (0.83–1.08)	0.95 (0.83–1.08)	0.95 (0.82–1.08)
Average number of residents per house (per 0.1 increase)	0.94 (0.88–1.01)	0.94 (0.88–1.00)	0.95 (0.89–1.02)
Proportion of rented households (per 10% increase)	0.95 (0.82–1.09)	0.94 (0.81–1.08)	0.90 (0.78–1.05)
Proportion of residents employed in health care services (per 10% increase)	1.01 (0.68–1.51)	1.00 (0.69–1.49)	1.01 (0.69–1.50)
Proportion of residents employed in agricultural industry (per 10% increase)	**0.80** (**0.67–0.95)**	**0.79** (**0.67–0.95)**	**0.77** (**0.65–0.91)**
Average number of people per bedroom (per 0.1 unit increase)	0.97 (0.83–1.13)	0.97 (0.83–1.14)	0.93 (0.79–1.10)
IRSD (per 100 units increase)	**0.66** (**0.54–0.79)**	**0.66** (**0.55–0.79)**	**0.62** (**0.51–0.74)**
	**Posterior mean of variance (95% CI)**		
Unstructured	0.00 (0.00–0.04)	–	0.17 (0.12–0.27)
Structured	0.41 (0.26–0.66)	0.43 (0.29–0.67)	–
	**Deviance information criterion (DIC)**		
	1275.270	1275.140	1301.080

**Fig. 4.**
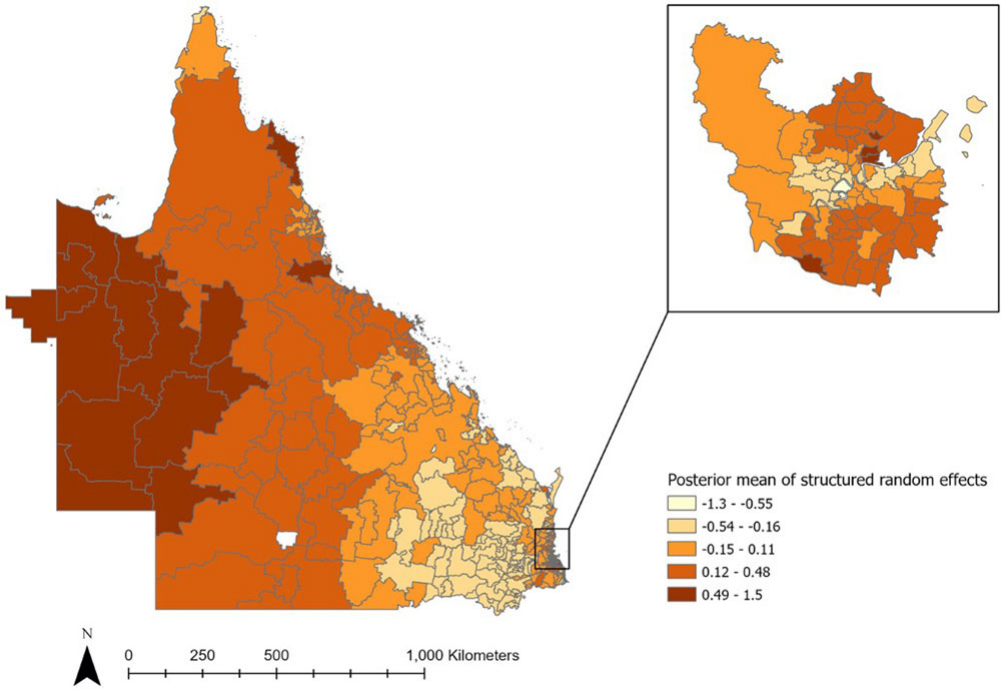
Posterior mean variance of structured random effects per postal area across Queensland.

Next, the posterior mean variance of spatially structured random effects per postal area was mapped (Fig. 4). After accounting for the studied community risk factors, hot spots were still largely detected in Outback and Far North Queensland, where a large proportion of these postal areas were classified as remote or very remote regions. A clear decreasing gradient of hot spots from very remote to remote to outer regional and to inner regional zones can be visualised. However, at the Brisbane urban city area, there was no distinct pattern in the distribution of hot spots.

There were 423 postal areas with residents in 2011. In the sensitivity analysis using census data and ESBL-Ec incidence from 2011, the spatially structured model (DIC = 897.462) similarly had the best performance. Better socioeconomic status within the communities (RR = 0.61, 95% CI 0.46–0.82, per 100 units increase) and higher proportion of residents employed in the agricultural industry (RR = 0.62, 95% CI 0.44–0.85, per 10% increase) were consistently protective factors of lower ESBL-Ec incidence (Supplementary Table S3).

## Discussion

The annual incidence rate of ESBL-Ec in Queensland, Australia, has increased by more than 5-fold between 2010 and 2019, with a sharper escalation noted between 2016 and 2018. The hot spot analysis identified that the distribution of ESBL-Ec cases changed from random to clustered after 2014, with the highest number of hot spots detected in 2016. This suggests that there are area-specific risk factors that escalated ESBL-Ec spread in some communities. ESBL-Ec hot spots were more prevalent in Outback and Far North remote Queensland regions, and interestingly, only few hot spots were identified in the Brisbane city region despite higher population density. The protective factors for lower ESBL-Ec incidence were better socioeconomic status and higher proportion of residents employed in agricultural industry within the community. A higher proportion of Aboriginal and Torres Strait Islander residents in the community was associated with higher ESBL-Ec incidence, but this was not statistically significant. Despite accounting for these demographics, residual spatially structured effects were still present, and were indicative of unaccounted ESBL-Ec clusters especially in the more remote communities but also within Brisbane city.

Interestingly, population and household density were not predictive of ESBL-Ec incidence. As the outcome data were collected from public health care facilities only, the number of ESBL-Ec cases from city areas was likely under-represented as access to private health care services is higher in the city. Nonetheless, a population-based prevalence study was recently conducted in the Netherlands investigating predictors of ESBL-producing Enterobacterales carriage as compared to non-carriers in the community. Consistent with our results, this study also reported that population density was not predictive of higher incidence [21]. Although a mathematical modelling study had suggested that the probability of ESBL-Ec transmission could be higher within denser households, transmission can be attenuated with good hand hygiene practices [22]. Good sanitation behaviour, such as proper handwashing, may have a bigger influence on preventing ESBL-Ec spread in the community than restricting overcrowding, and could be validated with further studies.

Next, our results indicate that the spread of ESBL-Ec in the community is likely not discriminative of age. Although a population-based study conducted in France reported that age greater than 65 years was predictive of urinary tract infection caused by ESBL-Ec [10], the study sample had only included infected patients who were likely with higher risk of being sick. On the other hand, our population-based sample included a substantial proportion (~22.4%) of ESBL-Ec carriers who did not develop subsequent infection, and are likely younger and at lower risk of infection. As such, although ESBL-Ec transmission in the community is likely not influenced by age, future public health measures to prevent spread should be targeted in elderly communities where infections are more likely to develop from carriage.

Our results also show that working in the agricultural industry may lower the incidence of ESBL-Ec in the community. This is in contrast to molecular evidence presented from several studies where farm animal to human transmissions were reported [9]. The population-based French sample had also observed poultry and pig densities in the community as predictors of urinary tract infection caused by ESBL-Ec [10]. There are several postulations to these discrepancies in observations. Firstly, our covariate measuring proportion of agricultural industry workers may be insufficiently valid in measuring spread of ESBL-Ec from farm animals to humans. The covariate measured people who were also employed in other agricultural practices, such as forestry, fruit and vegetable farming and fishing, which have shown no evidence as risk factors of ESBL-Ec sources. As a result, the true effect between contact with farm animals and ESBL-Ec incidence may have been masked. Alternatively, ESBL-Ec spread between farm animals and humans may not be significant within Queensland communities. Australian farming practices tend to be less intensive and are conservative in antibiotics use in food animal production [23]. In addition, there is evidence that ESBL-Ec isolates derived from food animals and humans are genetically distinct [24, 25], suggesting that colonisation in humans is highly unlikely to be acquired from this source. This molecular evidence, combined with our spatial epidemiological results, indicate that direct transmission of ESBL-Ec from farm animals contact to human may not be likely in Queensland communities and hence, does not present as a risk factor. To further validate the association between exposure to farm animals and ESBL-Ec incidence, future observational studies would require a more robust tool to measure farm exposure and results should ideally be complemented with molecular evidence.

Communities with higher proportion of Aboriginal and Torres Strait Islander residents were associated with higher incidence of ESBL-Ec, although this observation was not statistically significant in the multivariable analysis. The hot spots detected in Far North Queensland region (Fig. 3) are likely accounted by the high proportion of Aboriginal and Torres Strait Islander residents, which comprised between 30% and 95% of population there (Supplementary Fig. S3). Based on the multivariable analysis, the socioeconomic status associated with the indigenous population likely had an influence on risk of ESBL-Ec acquisition.

The mapping of posterior mean variance of spatially structured random effects shows that hot spots were more likely detected in remote areas, even after accounting for multiple demographic risk factors in this study. The remoteness index is defined by ABS based on relative access to services from urban centre or locality [26], and our results suggest that poor access to services may have an effect on increased ESBL-Ec incidence. As visualised in Figure 4, there is a clear decreasing gradient of hot spots from very remote to remote to outer regional to inner regional areas. This suggests the plausibility of a negative association between access to services and incidence of ESBL-Ec. Public health measures that aim to reduce the spread of ESBL-Ec should be targeted and tailored for the remote communities and future research should be planned to identify specific risk factors in remote settings. Interestingly, the decreasing gradient of hot spots across the remoteness index was not applicable to urban Brisbane city, as a substantial number of hot spots were detected here. As such, the risk factors for ESBL-Ec acquisition in cities are likely differential to remote and regional communities in Queensland.

One limitation in our ecological study is that there are likely other spatial factors that were not collected and accounted, such as local temperature. It was reported that the incidence of *E. coli* bloodstream infections increases with increased mean local temperature or over the summer season [27], postulated by changes in human behaviour, bacteria density in environmental sources or host immune function [28]. More specifically, this positive association with temperature was also observed with incidence of ESBL-producing bacteria in Germany [29]. In our hot spot and spatial analyses, we found that hot spots were more likely identified in Outback and Far North Queensland, which experience generally hotter weather conditions as compared to Brisbane city. Nonetheless, the dynamics between temperature and incidence of bacteria acquisition are extremely complex and multifactorial. Further epidemiological, spatial, time-series and molecular analyses are required to elucidate the relationship between ESBL-Ec incidence and temperature or seasonal changes. Another limitation in our study is that healthcare-associated risk factors, such as community consumption of antibiotics and hospital exposure, were not accounted in the analyses. While modelling studies have predicted that reducing overall antibiotic use could reduce drastically ESBL-producing bacteria colonisation [30], other population-based studies have reported that these predictors were not significantly associated with or had limited contribution to ESBL-Ec incidence in the community [10, 21]. These healthcare-associated risk factors may account for some of the residual spatially structured effects detected in the Brisbane city area, where accessibility to antibiotics and medical care is higher as compared to remote areas. In addition, the spatial analysis assumes that each acquisition event had occurred within the geographical boundary of the reported residential postal area, which is factually false since acquisition may have occurred elsewhere with a fluid population. We also could not quantify and differentiate between hospital-acquired and community-acquired ESBL-Ec cases with the available data. The presence of hospital-acquired ESBL-Ec cases in our study sample would have made our results less precise.

Nonetheless, this is the first study to describe ESBL-Ec clusters using hot spot analysis and to determine community risk factors using spatial analysis. Transmission of ESBL-Ec in the community is very dynamic and complicated, exacerbated by the fluidity of humans moving across diverse communities on a daily basis. We tried to elucidate risk factors that may promote spread and acquisition in people when ESBL-Ec bacteria are introduced into the community. While the results are derived from a population level, this study serves as good evidence to inform hypotheses for future observational studies.

## Conclusion

The annual incidence rate of ESBL-Ec increased five-fold from 2010 to 2019, with the highest escalation observed between 2016 and 2018. The distribution of cases was identified as random before 2014 and as clustered after 2014, indicating the presence of area-level specific risk factors that can enhance the spread of ESBL-Ec in the community. The hot spot analysis identified a higher number of clusters found in Outback and Far North Queensland regions, where postal areas are mostly classified as remote with low accessibility to services from urban centre or localities. As such, future public health measures may be targeted at these remote communities to reduce transmission and research should be additionally conducted to identify specific risk factors for transmission in these remote settings. The spatial multivariable analysis suggests that better socioeconomic status could be protective of lower ESBL-Ec acquisition. Moreover, it appears that having a larger proportion of the population employed in the agricultural industry, which was a proxy for exposure to farm animals in this study, did not increase the risk of higher ESBL-Ec incidence in the community. Nonetheless, there are several other spatial factors, such as local temperature and healthcare-associated risk factors, that were not accounted for and should be further investigated in future studies. On a local context, future research should be conducted to investigate if the hotter climatic conditions of Outback and Far North Queensland or if poor accessibility to services are associated with increased ESBL-Ec incidence in Australia.

## Data Availability

The data that support the findings of this study are available from AUSLAB (PJA Solutions Pty Ltd), Pathology Queensland. Restrictions apply to the availability of these data due to its sensitivity nature. Data are available from the authors with the permission of Pathology Queensland. The other census data that support the findings of this study are openly available in ABS at https://www.abs.gov.au/census/find-census-data and https://www.qgso.qld.gov.au/statistics/theme/population/population-estimates/regions.

## Supplementary material

For supplementary material accompanying this paper visit http://doi.org/10.1017/S0950268822001637. click here to view supplementary material
