# Supplementary material for: Incidence and predictors of Escherichia coli producing extended-spectrum beta-lactamase (ESBL-Ec) in Queensland, Australia from 2010 to 2019: a population-based spatial analysis
Source: Epidemiol Infect. 2022 Oct 26;150:e178. doi: 10.1017/S0950268822001637 (PMC9987021; doi:10.1017/S0950268822001637)
Supplement: Supplementary file 1 [file S0950268822001637sup001.docx]

**Supplementary Table 1. Description of collected demographic variables**

| **Demographic variable specific to place of usual residence** | **Description** | **Denominator (where applicable)** |
| --- | --- | --- |
| Number of people per kmsq (1000 pax) | Number of residents per km squared of the postal area | - |
| Median age | Median age | - |
| Proportion of male (10%) | Male | All residents, excluding tourists |
| Proportion of Aboriginal and Torres Strait Islander (10%) | Aboriginal and Torres Strait Islander | All residents, excluding tourists |
| Proportion of residents born in Australia (10%) | People who were born in Australia | All residents, excluding tourists |
| Average number of children per family (0.1) | Number of children aged 0 to 14 years old in a family | All families, including couple families with and without children, one parent families and other family compositions |
| Average number of residents per house (0.1) | Number of residents usually resident in an occupied private dwelling, including up to 3 residents that were temporarily away from household on census night | All occupied private dwellings |
| Proportion of rented households (nr 7.7%) | Households with rental arrangement | All occupied private dwellings |
| Proportion of residents employed in health care services (10%) | People employed in health care services, including hospital, medical, allied health, residential care and social assistance | All residents at least 15 years old and employed at time of census |
| Proportion of residents employed in agricultural industry (10%) | People employed in agriculture, forestry and fishing industries | All residents at least 15 years old and employed at time of census |
| Average number of people per bedroom (0.1) | Number of residents usually resident in an occupied private dwelling, including up to 3 residents that were temporarily away from household on census night | All bedrooms in each occupied private dwelling, including caravans |
| Index of relative socioeconomic disadvantage (IRSD) (100) | Measure of relative socioeconomic disadvantage, including low income households, dwellings without internet connection or car, residents with low or no educational attainment, unemployment, one-parent families with dependent, elderly with disability or long-term health condition, divorced or separated families, employment as Labourers, Machinery operators and drivers or low skill Community and Personal Service workers, overcrowded housing, people with poor English skills, rented dwellings with rent less than $215/week;  Lower score indicates higher relative socio-economic disadvantage | - |
| Proportion of residents with tertiary education | People aged above 15 years old with at least a Diploma or Bachelor for their highest educational attainment | All residents aged above 15 years old |
| Median income (personal) | Total income (including wages, pensions, superannuation, allowances, profits and any passive income) a person usually receives each week, for people aged at least 15 years old |  |

**Supplementary Table 2. Univariate analysis of demographic predictors and ESBL-Ec incidence in 2016**

| **Demographic variable (unit)** | **RR (95% CI)** |
| --- | --- |
| Number of people per km squared (per 1000 people increase) | 0.93 (0.88-0.99) |
| Median age (per 1 year increase) | 0.99 (0.98-1.00) |
| Proportion of male (per 10% increase) | 1.44 (1.05-1.96) |
| Proportion of Aboriginal and Torres Strait Islander (per 10% increase) | 1.25 (1.20-1.30) |
| Proportion of residents born in Australia (per 10% increase) | 1.02 (0.97-1.08) |
| Average number of children per family (per 0.1 unit increase) | 2.25 (1.91-2.65) |
| Average number of residents per house (per 0.1 increase) | 1.07 (1.03-1.11) |
| Proportion of rented households (per 10% increase) | 0.99 (0.97-1.01) |
| Proportion of residents employed in health care services (per 10% increase) | 1.17 (1.12-1.22) |
| Proportion of residents employed in agricultural industry (per 10% increase) | 1.26 (1.03-1.55) |
| Average number of people per bedroom (per 0.1 unit increase) | 0.95 (0.87-1.04) |
| Index of relative socioeconomic disadvantage (per 100 units increase) | 1.21 (1.13-1.29) |
| Number of people per km squared (per 1000 people increase) | 0.65 (0.60-0.69) |

**Supplementary Table 3. Multivariable spatial analysis of demographic predictors and ESBL-Ec incidence in 2011, with comparison of results across 3 different models**

| **Year 2011** | **Spatially unstructured random effects model (*s*+*µ*)** | **Spatially structured model (*s*)** | **Unstructured random effects model (*µ*)** |
| --- | --- | --- | --- |
| **Demographic variable** | **Relative risk (95% CI)** | | |
| Number of people per km squared (per 1000 people increase) | 0.96 (0.78-1.19) | 0.95 (0.77-1.17) | 1.00 (0.82-1.22) |
| Median age (per 1 year increase) | 1.00 (0.96-1.04) | 1.00 (0.96-1.04) | 1.00 (0.96-1.04) |
| Proportion of male (per 10% increase) | 0.67 (0.32-1.41) | 0.67 (0.31-1.40) | 0.81 (0.39-1.64) |
| Proportion of Aboriginal and Torres Strait Islander (per 10% increase) | 0.97 (0.73-1.29) | 0.97 (0.73-1.28) | 1.08 (0.83-1.41) |
| Proportion of residents born in Australia (per 10% increase) | 0.97 (0.78-1.20) | 0.97 (0.79-1.21) | 0.98 (0.81-1.18) |
| Average number of children per family (per 0.1 unit increase) | 0.90 (0.76-1.06) | 0.90 (0.76-1.06) | 0.90 (0.76-1.07) |
| Average number of residents per house (per 0.1 increase) | 1.02 (0.93-1.12) | 1.02 (0.93-1.12) | 1.02 (0.94-1.12) |
| Proportion of rented households (per 10% increase) | 0.94 (0.75-1.14) | 0.94 (0.75-1.15) | 0.94 (0.76-1.15) |
| Proportion of residents employed in health care services (per 10% increase) | 0.93 (0.51-1.69) | 0.93 (0.50-1.69) | 0.86 (0.50-1.49) |
| Proportion of residents employed in agricultural industry (per 10% increase) | 0.63 (0.46-0.84) | 0.62 (0.44-0.85) | 0.57 (0.41-0.77) |
| Average number of people per bedroom (per 0.1 unit increase) | 1.07 (0.76-1.46) | 1.06 (0.78-1.45) | 1.10 (0.80-1.52) |
| Index of relative socioeconomic disadvantage (per 100 units increase) | 0.62 (0.46-0.81) | 0.61 (0.46-0.82) | 0.68 (0.51-0.89) |
|  | **Posterior mean of variance (95% CI)** | | |
| Unstructured random effects | 0.00 (0.00-0.07) | - | 0.18 (0.08-0.35) |
| Spatially structured random effects | 0.36 (0.16-0.82) | 0.41 (0.20-0.87) | - |
|  | **Deviance information criterion (DIC)** | | |
|  | 898.168 | 897.462 | 907.401 |


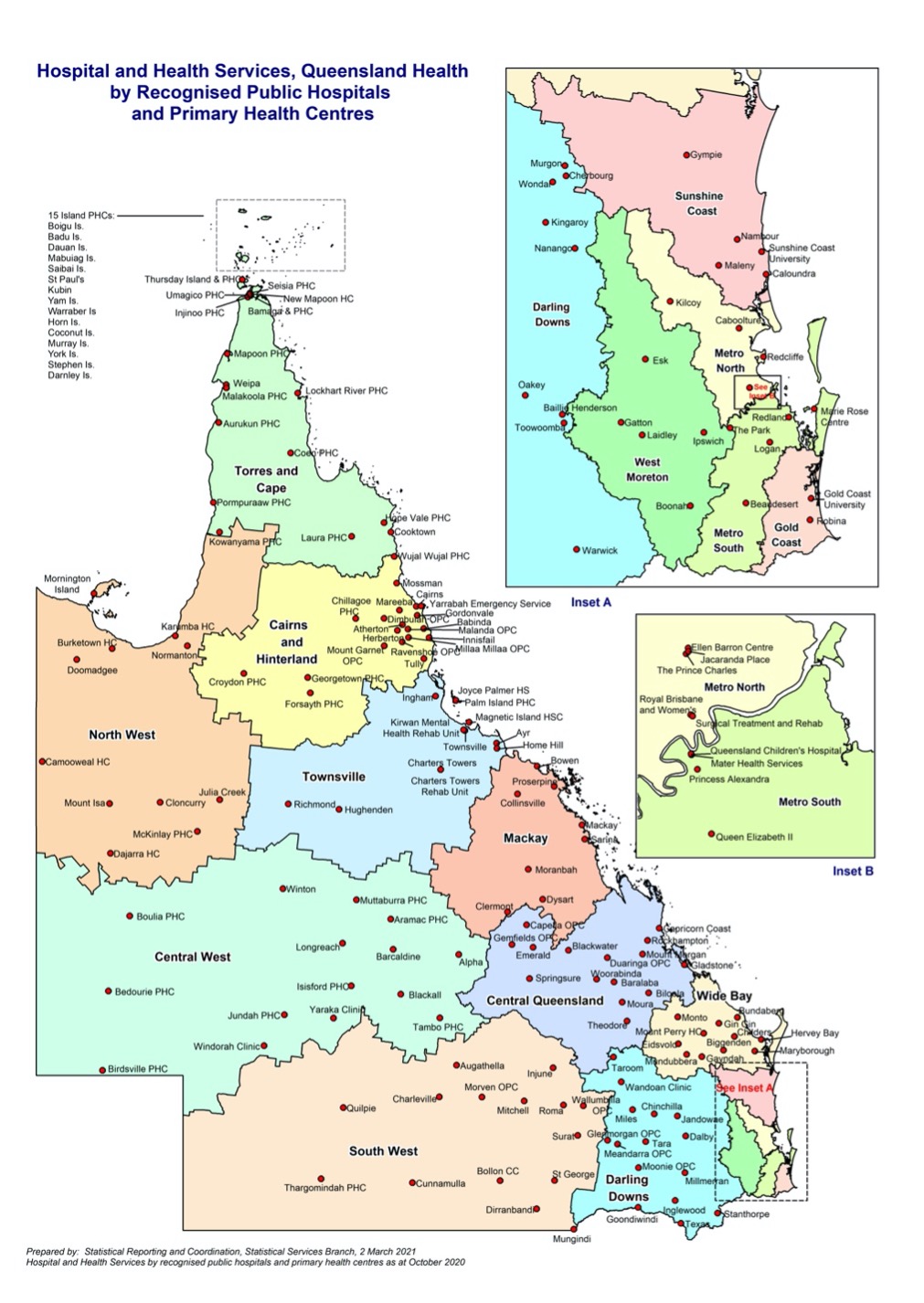


**Supplementary Figure 1.** 15 Hospital and Health Services in Queensland


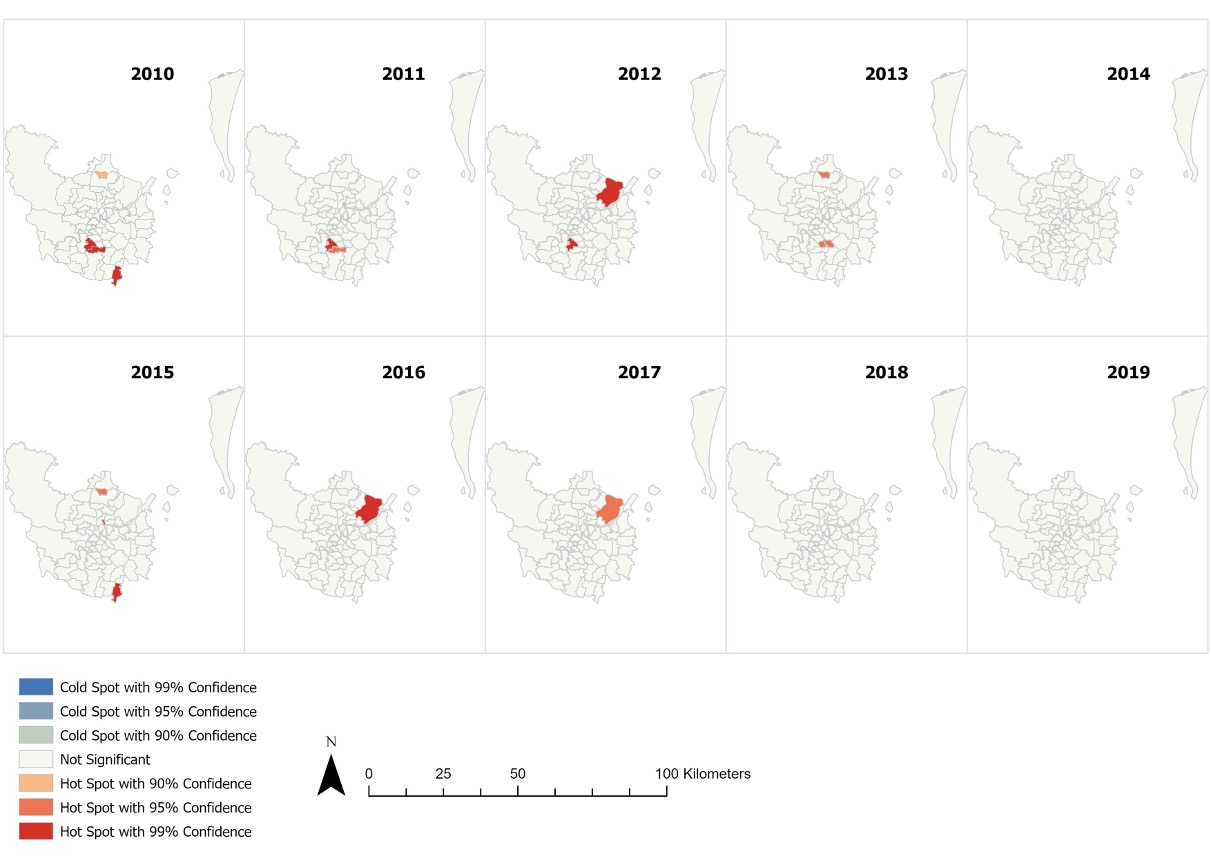


**Supplementary Figure 2. Annual ESBL-Ec hot spots identified in Brisbane**


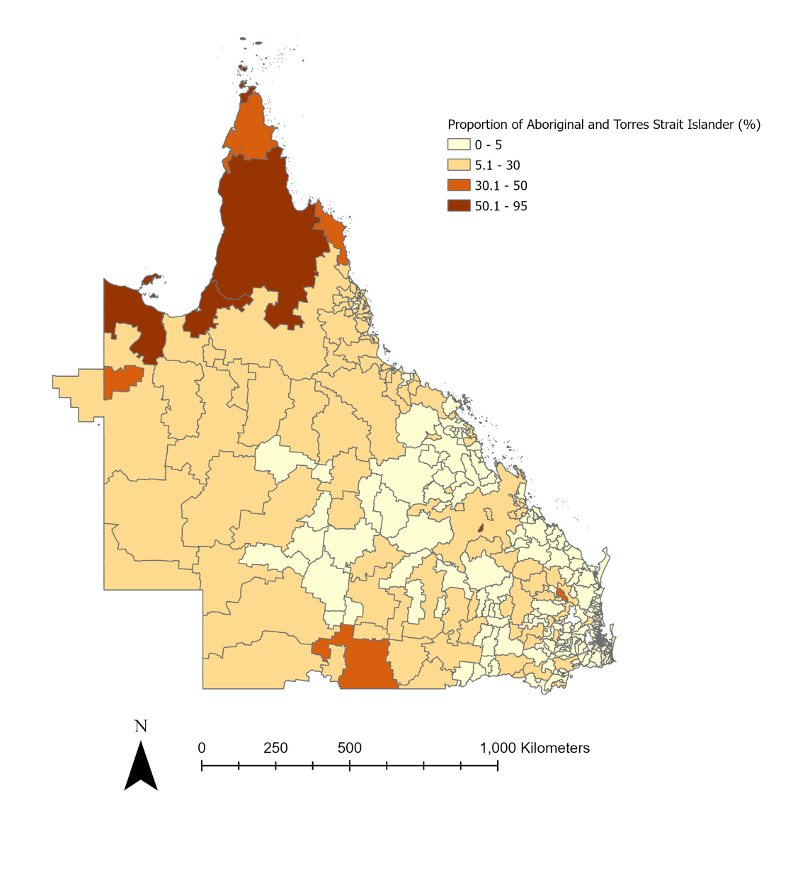


**Supplementary Figure 3. Proportion of Aboriginal and Torres Strait Islander residents per postal area across Queensland in 2016**
